# Hospital Meetings, &c.

**Published:** 1899-12-16

**Authors:** 


### HOSPITAL MEETINGS, &C.

### CHARING CROSS HOSPITAL SPECIAL APPEAL.

Over £50,000 Still Required.

A meeting of the Charing Cross Hospital Special Appeal Committee was held at the Mansion House on Wednesday, 6th instant, the Lord Mayor presiding. Notwithstanding that the fog and rain made the day a most uninviting one there was a considerable gathering, including several ladies. Among those present were the Lady Mayoress, Mrs. Beerbohm Tree, Mr. and Mrs. Albert Sandeman, Mr. Lund (secretary to the committee) and Mrs. Lund, Mrs. Wooley, Mr. J. H. Borrett (chairman of the hospital), Rev. Stephen Barrass (the Lord Mayor's chaplain), Lieut.-Colonel Probyn, Dr. Wilcocks, Mr. J. S. Pearce, Mr. H. H. Vivian, Mr. Bloxam, Mr. D. Laing, Mr. W. H. Chaplin, and Mr. P. B. Wooldridge (assistant secretary).

The report presented stated that since the special appeal was made in November, 1896, the total amount paid or promised was £56,753. Not only had old friends come forward to help but many new supporters had been interested in the cause. The City companies had, with their usual generosity, contributed together £2,936. The Ladies' Committee had organised dances and concerts, which had realised £1,103, while entertainments given by the directors of the Pavilion, Tivoli, Oxford, and Charing Cross music halls, as well as at Philbeach Hall, Kensington, produced more than £500. The Diamond Jubilee procession enabled the committee to make £1,000, by letting seats erected round the hospital. The great bazaar at the Royal Albert Hall resulted in the net profit of £15,000 being added to the appeal fund. During the past three years, owing mainly to a large sum received from legacies, not only had the ordinary expenditure for the maintenance of the hospital been met, but the debt of £17,000 had been cleared off. Further, the Council had purchased freeholds at a cost of £18,000, so that the whole of the triangular space on which the hospital stands, with the exception of that portion occupied by the Ophthalmic Hospital, was now available for the necessary improvements; invested £6,000, contributed definitely towards the endowment of beds; and paid £3,000 for the lease of the one house in Chandos Street not the property of the hospital. The Council had now in hand the sum of £22,000 towards the new hospital wing. The utmost pains had been taken to ascertain what alterations were needed in order that the hospital might be fully equipped for its work, the committee being assured that the public would expect that the new hospital when completed should be a model of what a hospital of its size should be. Mr. A. Saxon Snell, having submitted the set of designs which was awarded the first place in a limited competition, had been appointed architect. Full detailed plans had been prepared, and the building would be commenced without delay. The total needed for the improvements was now estimated to be at least £75,000. Towards this the council had £22,000 in hand from the Appeal Fund, and they hoped that individual donors might be found to take up some one or other of the special departments as a single donation as had been already done in the case of many other hospitals. But making allowance for such gifts, they felt that they must still address themselves to the task of raising at least £50,000 before the good work would be accomplished.

The Chairman said that when asked to preside at that meeting he had no hesitation in accepting, because from very early times Charing Cross Hospital had always had his warmest sympathy. He looked on it as a harbour of refuge for any urgent case. There was no red tape—the mere fact of a case being urgent ensured immediate attention. He then proceeded to recapitulate and emphasise the points in the report, and concluded by saying he felt sure it was not asking too much to appeal for the £50,000 still needed for such an institution, with its past history of help and succour to suffering humanity and its promise of beneficence to future generations. Charing Cross Hospital was always with us, and we must not forget its claims in the more exciting events through which we were passing.

Lord Glenesk moved the adoption of the report. He said the hospital as it now stood was not fit either for the patients or for the doctors who attended them. Except, perhaps, the War Office in Pall Mall, he thought it was the worst equipped public building in London. It was altogether too small for the purposes of to-day. It met the requirements of 30 years ago, but not those of the present day, and it behoved the honour of the nation that this should be put right. Far better was the system of maintaining hospitals by voluntary subscriptions and individual generosity than any system of State regulation, with its consequent unnecessary uniformity of plan and organisation. As they stood, our hospitals were public monuments of English kindness. The Charing Cross Hospital had been relieved of a debt of £17,000, and had paid £18,000 for the freehold of the houses adjoining. This had given them an island of their own, and on that island they were going to erect a model hospital. For that purpose they were appealing for support, and he had no fear for the result. In one hospital with which he was connected they had considered themselves behind the times; they had boldly incurred the expense of the improvements they considered necessary, and they had had no difficulty whatever in obtaining the money to pay for them. Proceeding, the speaker gave instances of the great benefits derived, at the institution of which he was speaking, both by the patients and by the surgeons, from the improvements effected. He relied, he said, on the boundless generosity of the British public, who seemed never tired of good works. He thought there was no danger of the sources drying up; for a good cause they could rely on a generous response. That Special Appeal, fo instance, was started in the year when the Prince of Wales made his appeal for the hospitals. They asked for £100,0 at the rate of £20,000 a year, and in that very year they got £26,000. In his official capacity at the Mansion House the Lord Mayor would know how nobly appeals to public generosity were met. The money was wanted to enlarge and perfect Charing Cross Hospital, to enable it to take all the cases that came to it, and to give the patients the benefits of good air and of the latest scientific improvements. He was confident they would get what was required, and be able to erect a building in the centre of London which should be its honour and its pride.

The Rev. Prebendary Kitto, in seconding the motion, said that at the outset the question had been raised whether they were not making this appeal at an unfavourable time, but in his experience the favourable moment never did come; the right plan was to go for what was wanted and wait results, and that plan in the case of the Charing Cross Hospital had been very successful. It was no longer in a state of hopeless insolvency, and they had a substantial sum towards the building fund. And what was very satisfactory was that the ordinary income of the hospital had been more than fully maintained. It showed that the more people gave the more they liked to and the more their sympathies were drawn out. He felt sure that the liberality of the British public would not be limited either to the voluntary half million which was being contributed to the War Fund or to the involuntary contributions which the Chancellor of the Exchequer would later on impose. He felt quite confident that as the money was wanted it would come in.

Mrs. Beerbohm Tree proposed the next resolution: "That this meeting gratefully acknowledges the liberal support which has been given to the hospital, and pledges itself to do all in its power to raise the amount required to complete the improvements." She said: To my lots falls the pleasant duty of thanking the public for what they have already done, and it also devolves upon me to point out that this gratitude of ours takes the form of that "keen sense of favours to come" of copy-book fame. Therefore, when I have said, "Thank you," it is not quite in the character of one who exclaims, "Hold, enough!" I am reminded of the Scotch shepherd who complained to the steward of his laird, "Och, but that master of yours is about the meanest mon I ever knew!" "Mean," answered the bewildered steward. "Och, the varra meanest mon I ever met." "But how?" "Why, when he'd been chasin' the deer in the mountain he invited me take a glass of whisky; as he was pourin' it into the glass I said, 'Stop! Stop!'—and he stopped!" (Laughter.) Well, if the glass is as yet but half full of the whisky of your bounty, let no polite but insincere expression of modest protest on my part prevent you from pouring out your generosity until the glass is full—nay, brimming over. We are a nation of enthusiasts, and we must be content with the drawbacks of our besetting virtue. At the present time the entire philanthropy of the nation has focussed itself upon the thought of alleviating the sufferings and anxieties of those brave and beloved soldiers who are fighting for us in South Africa. (Cheers.) Splendid as is the outburst of enthusiasm and loyalty which has found expression in pity and solicitude for those dear to our "gentlemen in khaki ordered South," we ought, perhaps, to pause while we scatter our benevolence to give a thought to those sick and wounded who are lying at our very gates, and who may be fighting the battle of life as bravely at home as our soldiers are fighting the battle of the Empire abroad. "Charity is the scope of God's commands," as St. Chrysostom said, and those commands, while they urge us to stretch out one hand to the thousands who are far away, surely bid us extend the other to the millions close at hand. (Cheers.) The hospital is what its name denotes—it is the abiding place of our guests. The inmates of the hospital—the wounded, the maimed, the sick, the convalescent, the dying—are the guests of the nation; of those of it who are strong, who are whole, who are long-lived, who are well-to-do. Let us receive these our guests, then, in the true spirit of hospitality, denying them no comfort, withholding no courtesy; making glad their days, soothing them tenderly to rest when the night comes. This, the Charing Cross Hospital, is our guest-house, whose welcoming gates we want to widen to the sad and eager crowd waiting to enter. You have but to give again as generously and as much as you have already given and our heart's desire is fulfilled. I will conclude in the words of a forgotten poet:—

"Give, as the morning that flows out of heaven,
Give, as the waves when their channel is riven,
Give, as the free air and sunshine are given,
Lavishly, utterly, carelessly give.
Not the waste drops of your cup overflowing,
Not the faint sparks of your heart overglowing,
Not a pale bud from June roses blowing,
Give as He gives you Who gave you to live.
Pour out your love like the rush of a river,
Wasting its waters for ever and ever,
Through the burnt sands that reward not the giver,
Lavishly, utterly, carelessly give."

(Cheers.)

Alderman and Sheriff Treloar seconded the resolution, which was supported by

Mr. Stanley Boyd, of the hospital surgical staff. Their difficulties had been increased, he said, by the fact that the London County Council—no doubt quite properly from their point of view—had insisted on their having another staircase, with the result that there was direct communication between the out-patients' department and the wards, and every now and again scarlet fever and other germs landed among the in-patients with dire results. Then the science of surgery had advanced so enormously that operations which not many years ago were never dreamed of were now of daily occurrence. The recovery of patients was now much more rapid, so that a far greater number was treated than used to be the case. All these things meant a great increase in the tax on the staff, and it was only right that proper accommodation should be provided both for the medical and the nursing staff. A hospital could not stand still, but must always be adding to its appliances if it was to do the best that could be done for its patients. He was sure no money was better invested than what was placed in the hands of the treasurer of one of the great London hospitals or convalescent homes.

The Rev. Simeon Singer, as a Jewish Rabbi, testified to the unsectarian character of the hospital and the tender regard shown by everybody connected with the institution to the religious scruples of members of his own persuasion. England was the only country where there was no specific Jewish hospital, and so long as the hospitals were conducted in the spirit of that at Charing Cross there was no fear of a sectarian hospital being started.

The resolution was adopted unanimously, and a vote of thanks to the Lord Mayor brought the meeting to a close.

### LONDON HOSPITAL.

The quarterly general Court of Governors of the London Hospital was held on Wednesday, 6th inst., Mr. J. H. Hale presiding.

The report of the House Committee, which was presented and adopted, dealt in detail with the various temporary alterations in arrangements necessitated by the carrying out of the improvements in the hospital already decided upon, and it was mentioned that £25,000 had been received anonymously toward the expense of the out-patient department to be erected outside the present buildings. The committee reported that the various improvements had made good progress. With regard to the pathological department, the Sir Andrew Clark Memorial Committee had decided to hand over all the funds collected for the purpose of erecting a memorial to the late distinguished physician to the governors of the hospital, and this money would be applied toward meeting the expenses of the erection of the pathological department, the building to bear the name of the "Sir Andrew Clark Memorial."

Tenders had been accepted for the erection of an isolation block on the ground recently purchased at a cost of £22,024, and the work would be put in hand at once. A very great need of the hospital would thus be met, the inconveniences at present felt through the need of such a block creating great difficulty in carrying on the work.

The Princess of Wales on a recent private visit took the utmost interest in the work being carried on. Her Royal

Highness had most generously presented the hospital with a complete set of instruments needed for applying a new cure for lupus, recently introduced in Copenhagen, by light. As soon as the electricity needed could be supplied—probably by the end of the year—the system was to be started in a part of the temporary Harrison ward, partitioned off for the purpose. Dr. Stephen Mackenzie had paid a visit to Copenhagen for the purpose of personally seeing all the details of the work, and he would take the medical direction of the department. Two members of the nursing staff had also been sent to Copenhagen and thoroughly trained.

The executors of the late Baroness de Stern, having determined to transfer a bequest of £60,000 made by her to charities to the London Hospital for the erection and endowment of a convalescent home, a site had been purchased for £1,270 at Tankerton, where a new home would be erected. £18,661 was reported as having been received in legacies and gifts during the quarter. In consequence of the death of Mr. J. H. Crossman, sen., one of the vice-presidents of the hospital, it had been decided to recommend that his brother, Mr. Alexander Crossman, be elected in his place. Dr. Turner, one of the physicians, having resigned, the necessary steps for filling his place were being taken, and also to elect him as a consulting physician. The resignation of Sister Buxton, for 37 years sister in the children's ward, was also reported with regret. In conclusion, the committee desired to draw special attention to the following statement: "The work at this hospital continues to be as heavy as ever, and now that letters have been abolished the numbers coming to the receiving room are very much increased. Unfortunately patients are very apt to flock to the hospital at precisely the same hour as they have been in the habit of attending at the outpatient department, and the committee will be very thankful if the governors will endeavour to make it known amongst the patients whom they may be sending that it would be much more convenient not to attend between the hours of twelve and two, when so many casualty cases come in, but that the physicians have more time to attend to such cases if they attend at other hours." The meeting terminated with the customary votes of thanks.

### THE SURGICAL AID SOCIETY.

On Monday, the 11th inst., the thirty-seventh annual meeting of the Surgical Aid Society was held at the Cannon Street Hotel, under the presidency of the Lord Mayor. In a few introductory remarks his Lordship expressed his gratification at being able to follow the general example of his predecessors in office, as the support of the Chief Magistrate recommended an important charity to the business men of the City. He congratulated the society on the extension of its premises, and on the increase in its revenue, but he pointed out that, with the advance in science and surgery, the beneficent work of the Surgical Aid Society must of necessity be extended to keep pace with it.

The Secretary read extracts from the report, of which copies were supplied to those present. Attention was drawn to the financial aspect of the past year's work; the income amounted to £17,157, an increase of £3,070. Of this sum it is gratifying to note that £705 is in additional annual subscriptions. Appliances to the number of 25,976 have been granted during the twelve months. This is, as one of the later speakers stated, at the rate of 500 a week, or 80 a day. The committee has found the present system of special grants very valuable, particularly in enabling patients to obtain the more costly appliances. The great event of the year has been the opening of the enlarged premises, on July 13th, by the Earl of Aberdeen. The committee, having secured the adjacent buildings to its offices in Salisbury Square, has been able to double the ground floor accommodation. The fitting-rooms, formerly in the basement, now communicate directly with the surgeons' rooms and with the central store room. The women's waiting-room has been enlarged, and a men's waiting-room provided. The local branches in the provinces are also gaining popularity, and two new branches have been established, the one at Plymouth, and the other Bristol. By the death of Sir Stuart Knill the society lost a much-regretted vice-president. Mr. Francis A. Bevan, J.P., D.L., has kindly accepted this office.

The Lord Mayor submitted to the meeting the resolution accepting the report, and pledging it to support the society. He pointed out strongly the sympathy accorded to the society by the public, and the great benefit afforded to poor individuals and homes by it. "Poverty and suffering," he said, "must always be with us, but in no century had science conferred such incalculable benefits upon society. Science was, however, powerless to aid unless supplemented by the necessary, and sometimes costly, appliances. Their charity discouraged pauperisation, the recipients of its aid were in the proud position of helping themselves, whilst the surgeons appointed to examine the patients safeguarded the charity against imposition."

Mr. Ernest Flower, M.P., seconded the resolution. He remarked that his position on the committees of two hospitals enabled him to speak from experience of the record of good work well done by the society.

Mr. F. A. Bevan proposed a resolution of thanks to Mr. William Gray, who has been treasurer of the society for twenty years. "No amount of money contributed by the charitable for the work," he remarked, "could make the men to carry it on."

Archdeacon Sinclair, in the absence of the Bishop of London, proposed the reappointment of the committee, and the thanks of the meeting to the members of it.

All resolutions were carried unanimously, and the meeting closed with a vote of thanks to the Lord Mayor for presiding.

